# A New Free-Standing Aqueous Zinc-Ion Capacitor Based on MnO_2_–CNTs Cathode and MXene Anode

**DOI:** 10.1007/s40820-019-0301-1

**Published:** 2019-08-26

**Authors:** Siliang Wang, Qiang Wang, Wei Zeng, Min Wang, Limin Ruan, Yanan Ma

**Affiliations:** 1 Key Laboratory of Intelligent Computing and Signal Processing, Ministry of Education, Anhui University, No. 3 Feixi Road, Hefei, 230039 Anhui Province People’s Republic of China; 2 National Engineering Research Center for Agro-Ecological Big Data Analysis and Application, School of Electronics and Information Engineering, Anhui University, No. 111 Jiulong Road, Hefei, 230601 Anhui Province People’s Republic of China; 3 School of Sciences, Hubei University of Automotive Technology, No. 167 Checheng West Road, Shiyan, 442002 Hubei Province People’s Republic of China

**Keywords:** Energy storage, Zinc-ion capacitor, Battery-type and capacitor-type electrodes, MXene, Electrochemical performance

## Abstract

Restricted by their energy storage mechanism, current energy storage devices have certain drawbacks, such as low power density for batteries and low energy density for supercapacitors. Fortunately, the nearest ion capacitors, such as lithium-ion and sodium-ion capacitors containing battery-type and capacitor-type electrodes, may allow achieving both high energy and power densities. For the inspiration, a new zinc-ion capacitor (ZIC) has been designed and realized by assembling the free-standing manganese dioxide–carbon nanotubes (MnO_2_–CNTs) battery-type cathode and MXene (Ti_3_C_2_T_*x*_) capacitor-type anode in an aqueous electrolyte. The ZIC can avoid the insecurity issues that frequently occurred in lithium-ion and sodium-ion capacitors in organic electrolytes. As expected, the ZIC in an aqueous liquid electrolyte exhibits excellent electrochemical performance (based on the total weight of cathode and anode), such as a high specific capacitance of 115.1 F g^−1^ (1 mV s^−1^), high energy density of 98.6 Wh kg^−1^ (77.5 W kg^−1^), high power density of 2480.6 W kg^−1^ (29.7 Wh kg^−1^), and high capacitance retention of ~ 83.6% of its initial capacitance (15,000 cycles). Even in an aqueous gel electrolyte, the ZIC also exhibits excellent performance. This work provides an essential strategy for designing next-generation high-performance energy storage devices.

**Electronic supplementary material:**

The online version of this article (10.1007/s40820-019-0301-1) contains supplementary material, which is available to authorized users.

## Highlights

- A new zinc-ion capacitor (ZIC) was realized by assembling the free-standing manganese dioxide–carbon nanotubes (MnO_2_–CNTs) battery-type cathode and MXene (Ti_3_C_2_T_*x*_) capacitor-type anode in an aqueous electrolyte.
- The large specific capacitance of the MXene anode avoids the mismatch in capacitance between the cathode and anode of the ZIC.
- The superior performance of the proposed ZIC makes it a promising candidate for the next-generation energy storage devices.

**Electronic supplementary material:**

The online version of this article (10.1007/s40820-019-0301-1) contains supplementary material, which is available to authorized users.

## Graphical abstract

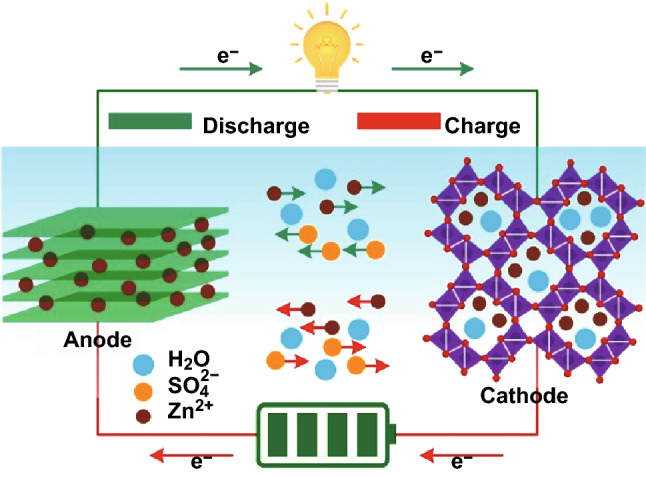

## Introduction

The issues of energy depletion and greenhouse effect owing to the over-consumption of non-renewable resources urgently require alternative green energy and efficient energy storage devices [1–3]. Currently, batteries (e.g., lithium-ion batteries, alkaline zinc–manganese batteries, and lead–acid batteries) and supercapacitors are the main energy storage devices [4–7]. Owing to their clear advantages (e.g., higher power density, longer cycle life, better cycle stability, and higher safety), supercapacitors are more promising energy storage devices compared with batteries [8–10]. However, restricted by their energy storage mechanisms, supercapacitors possess low energy density [11, 12]. To improve energy density without sacrificing the power density of supercapacitors, constructing ion capacitors, such as lithium-ion or sodium-ion capacitors, is possible. In the ion capacitors, battery-type electrodes and capacitor-type electrodes are paired in electrolytes [13–18]. For example, in the lithium-ion capacitor, the lithium-ion battery-type anode (cathode) contributes a high capacity using the lithium-ion insertion/extraction reaction, while the capacitor-type cathode (anode) provides high power by rapid ion adsorption/desorption. Therefore, lithium-ion capacitors possess higher energy density than supercapacitors and higher power density than batteries [16, 17]. Although considerable progress has been made in the area of lithium-ion and sodium-ion capacitors, the following drawbacks severely restrict their practical application [14, 15]. First, most lithium-ion and sodium-ion capacitors contain organic electrolytes that are flammable, volatile, and toxic. Second, the kinetics and capacitance mismatch owing to the sluggish kinetics of lithium-ion and sodium-ion battery-type electrodes and low specific capacitance of capacitor-type electrodes in organic electrolytes bring huge difficulties in implementing high-performance devices. Finally, determined by the intrinsic properties of lithium-ion and sodium-ion battery-type electrodes, the cycle life of these ion capacitors remains low. Therefore, employing new ion capacitor containing aqueous electrolytes that are safe and have long cycle life is essential.

Currently, rechargeable aqueous zinc-ion batteries have attracted considerable attention for their high capacity, fast kinetics, and high safety [19–22]. These unique properties of zinc-ion batteries give us an inspiration for designing novel zinc-ion capacitors (ZIC) that is composed of zinc-ion insertion/extraction battery-type cathodes and suitable capacitor-type anodes. Many materials (e.g., such as manganese-based oxides, vanadium-based oxides, Prussian blue analogs, Chevrel phase compounds, and polyanion compounds) have been used as the cathodes in zinc-ion batteries [23, 24]. Among these materials, manganese-based oxides, such as manganese dioxide (MnO_2_), have unique advantages of natural abundance, low toxicity, low cost, and multiple valence states of Mn [20, 21], which makes them potential battery-type cathode materials for ZIC.

Activated carbon (AC) is commonly used for a capacitor-type electrode in lithium-ion and sodium-ion capacitors [16, 25, 26]. However, using AC for capacitor-type electrode has following two defects: (1) AC-based electrodes exhibit limited capacity owing to the energy storage mechanism of electrochemical double-layer capacitors. (2) AC-based electrodes require the binder and conductive-additive, which increases the weight of the electrodes and decreases the final specific capacitance of the devices. Therefore, AC is unsuitable for the high-performance ZIC. MXene, a new type of two-dimensional (2D) layered materials with the formula of M_*n*+1_X_*n*_T_*x*_ (M represents the early transition metal, X represents carbon or nitrogen, and T_*x*_ represents F, O, and OH surface termination, *n* = 1, 2, 3), is extensively used in the field of energy storage owing to its superior properties of high conductivity, hydrophilic surface, and energy storage mechanism of intercalation/de-intercalation pseudo-capacitance [27–29]. Based on the properties and applications of MXene, we believe that MXene is a suitable capacitor-type electrode material for the ZIC.

Herein, we designed and realized an aqueous ZIC based on free-standing manganese dioxide–carbon nanotubes (MnO_2_–CNTs) battery-type cathode and Ti_3_C_2_T_*x*_ (as a representative of the MXene family) capacitor-type anode. In summary, the aqueous ZIC has the following advantages compared with the state-of-the-art lithium-ion and sodium-ion capacitors based on organic electrolytes: (1) The use of aqueous liquid or gel electrolytes does not pose any serious safety issues. (2) The use of MXene capacitor-type anode (intercalation/de-intercalation energy storage mechanism) eliminates the above-mentioned mismatch in kinetics and capacitance. (3) Compared with organic electrolytes, aqueous electrolytes are more stable during the charge–discharge cycles, which contribute to the long cycle life of the ZIC. As a proof of concept, the ZIC in an aqueous liquid electrolyte exhibits a high specific capacitance of 115.1 F g^−1^ (scan rate of 1 mV s^−1^), a high energy density of 98.6 Wh kg^−1^ (power density of 77.5 W kg^−1^), a high power density of 2480.6 W kg^−1^ (energy density of 29.7 Wh kg^−1^), a high capacitance retention of ~ 83.6% of its initial capacitance after 15,000 cycles, and a high Coulombic efficiency of above 93.3% during the cycles. Even in an aqueous gel electrolyte, the ZIC also exhibits an excellent performance. This study provides an effective way to design next-generation energy storage devices exhibiting high energy and power densities, excellent cycle stability, long life, and high safety.

## Experimental Section

### Reagents and Materials

Carbon nanotubes (CNTs, multi-walled, diameter ≤ 8 nm, length = 0.5–2 μm) were purchased from Beijing Boyu Gaoke New Material Technology Co., Ltd. Manganese acetate tetrahydrate [Mn(CH_3_COO)_2_·4H_2_O], ammonium persulfate [(NH_4_)_2_S_2_O_8_], 1-octanol, manganese sulfate monohydrate (MnSO_4_·H_2_O), hydrochloric acid (HCl), sodium dodecylbenzenesulfonate (SDBS), gelatin, and borax were purchased from Sinopharm Chemical Reagent Co., Ltd. Lithium fluoride (LiF) and zinc sulfate heptahydrate (ZnSO_4_·7H_2_O) were purchased from Aladdin Industrial Corporation and Sahn Chemical Technology (Shanghai) Co., Ltd., respectively.

### Fabrication of Free-Standing MnO_2_–CNTs Electrodes

MnO_2_ nanowires (NWs) were prepared by a method similar to our previous work [30]. CNTs, MnO_2_ NWs, and SDBS with appropriate weight ratios were mixed and put into deionized water (30 mL). The mixture was probe ultrasonicated for 30 min to form a homogeneous suspended solution. The prepared suspended solution was filtered through a membrane (pore size of 450 nm). After the filtered cake was dried and peeled off, a free-standing MnO_2_–CNTs electrode was obtained.

### Fabrication of Free-Standing MXene Electrodes

The MXene (Ti_3_C_2_T_*x*_) nanosheets were prepared according to our previous work [31, 32]. Briefly, LiF (1 g) was added to HCl (9 M, 20 mL), followed by magnetic stirring until LiF completely dissolved. Ti_3_AlC_2_ (1 g) was gently added to the above solution and magnetically stirred for 24 h (35 °C). After etching, the mixture was centrifuged several times. After centrifugation, the precipitate was re-dispersed in deionized water and sonicated for 1 h (Ar atmosphere below 35 °C) followed by centrifugation. Finally, the dark green supernatant of MXene nanosheets was achieved. The free-standing MXene electrodes were fabricated by directly filtering the MXene solution through a membrane (pore size of 450 nm) followed by drying and peeling off the filtered cake of MXene.

### Fabrication of Aqueous Liquid and Aqueous Gel Electrolytes

To prepare the aqueous liquid electrolyte, ZnSO_4_·7H_2_O (23 g) and MnSO_4_·H_2_O (0.676 g) were dissolved in deionized water (29.8 mL) and stirred until the solution was clarified. To prepare the aqueous gel electrolyte, gelatin (4.0 g) and borax (0.4 g) were added to deionized water (29.8 mL) and continuously magnetically stirred at 80 °C. After gelatin completely dissolved, ZnSO_4_·7H_2_O (23 g) and MnSO_4_·H_2_O (0.676 g) were added to the solution, and the stirring continued until a homogeneous solution was formed.

### Assembling the ZIC

The free-standing MnO_2_–CNTs and MXene films were cut to suitable sizes. The aqueous liquid ZIC was prepared by assembling the free-standing MnO_2_–CNTs cathode (pre-activated at a current density of 0.256 A g^−1^ for three charge–discharge cycles) and MXene anode with a liquid electrolyte-soaked (2 M ZnSO_4_ and 0.1 M MnSO_4_) separator in between. After changing the liquid electrolyte to gel electrolyte, the quasi-solid ZIC was prepared by the fabrication process similar to that for the aqueous liquid ZIC.

### Characterization

The morphology, structure, and composition of the samples were investigated using scanning electron microscopy (SEM, S-4800), transmission electron microscopy (TEM, JEM-2100), and X-ray diffraction (XRD, SmartLab, 9 KW). The electrochemical performances of the MnO_2_–CNTs cathode and MXene anode, such as cyclic voltammograms (CV), galvanostatic charge–discharge (GCD), and electrochemical impedance spectrum (EIS), were conducted on an electrochemical workstation (CHI660E) using metallic zinc foils as a counter-electrode and 2 M ZnSO_4_ and 0.1 M MnSO_4_ as an electrolyte in an electrolytic cell. The electrochemical performances of the ZIC in aqueous liquid and gel electrolyte were tested using a two-electrode system. The mass ratio of the MnO_2_–CNTs cathode to MXene anode was 1:2.14 according to the charge balance between the cathode and anode.

## Results and Discussion

**Fig. 1:**
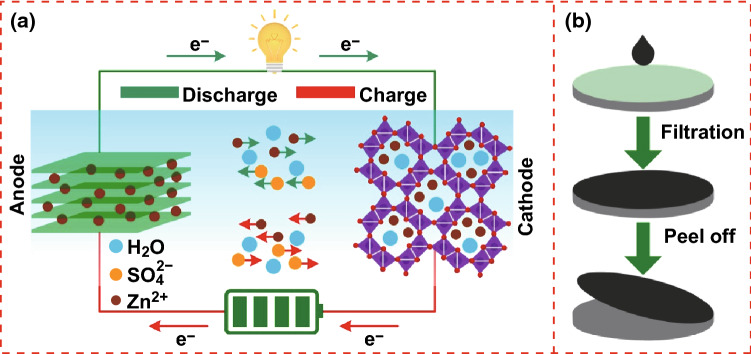
Schematic diagram of the ZIC and fabrication procedure of the free-standing electrodes, **a** schematic diagram of the ZIC: MnO_2_–CNTs serve as the cathode, MXene serves as the anode, and 2 M ZnSO_4_ and 0.1 M MnSO_4_ serve as the electrolyte; **b** fabrication procedure of the free-standing MnO_2_–CNTs cathode and MXene anode

The schematic diagram of the ZIC is shown in Fig. 1a. The ZIC consists of a MnO_2_–CNTs battery-type cathode (pre-activated by charge–discharge cycles), MXene capacitor-type anode, and 2 M ZnSO_4_ and 0.1 M MnSO_4_ electrolyte. In the steady state, the energy storage mechanism of the ZIC can be described briefly as follows. During charging, the zinc ions are extracted from the tunnels of MnO_2_ into electrolyte and subsequently intercalate into the interlayer of MXene. During discharging, the zinc ions de-intercalate from MXene into electrolyte and subsequently insert into the tunnels of MnO_2_. Both battery-type and capacitor-type energy storage mechanisms such as zinc-ion insertion/extraction (MnO_2_) and intercalation/de-intercalation (MXene) result in high energy and power densities of the ZIC. MnSO_4_ with an appropriate concentration was added to effectively inhibit the dissolution of MnO_2_, which significantly improved the cycle life of the ZIC. Figure 1b shows the fabrication procedure of the free-standing MnO_2_–CNTs cathode and MXene anode. To prepare the MnO_2_–CNTs electrode, MnO_2_ nanowires (NWs) and CNTs were probe sonicated for 30 min. Then, the well-mixed solution was vacuum-filtered through a membrane followed by vacuum drying. Finally, free-standing MnO_2_–CNTs film was peeled off from the membrane. A similar method was adopted to prepare a free-standing MXene electrode. Because the MXene solution with high dispersion can be obtained by liquid exfoliation, the solution was directly vacuum-filtered and did not require the step of probe sonication.

**Fig. 2:**
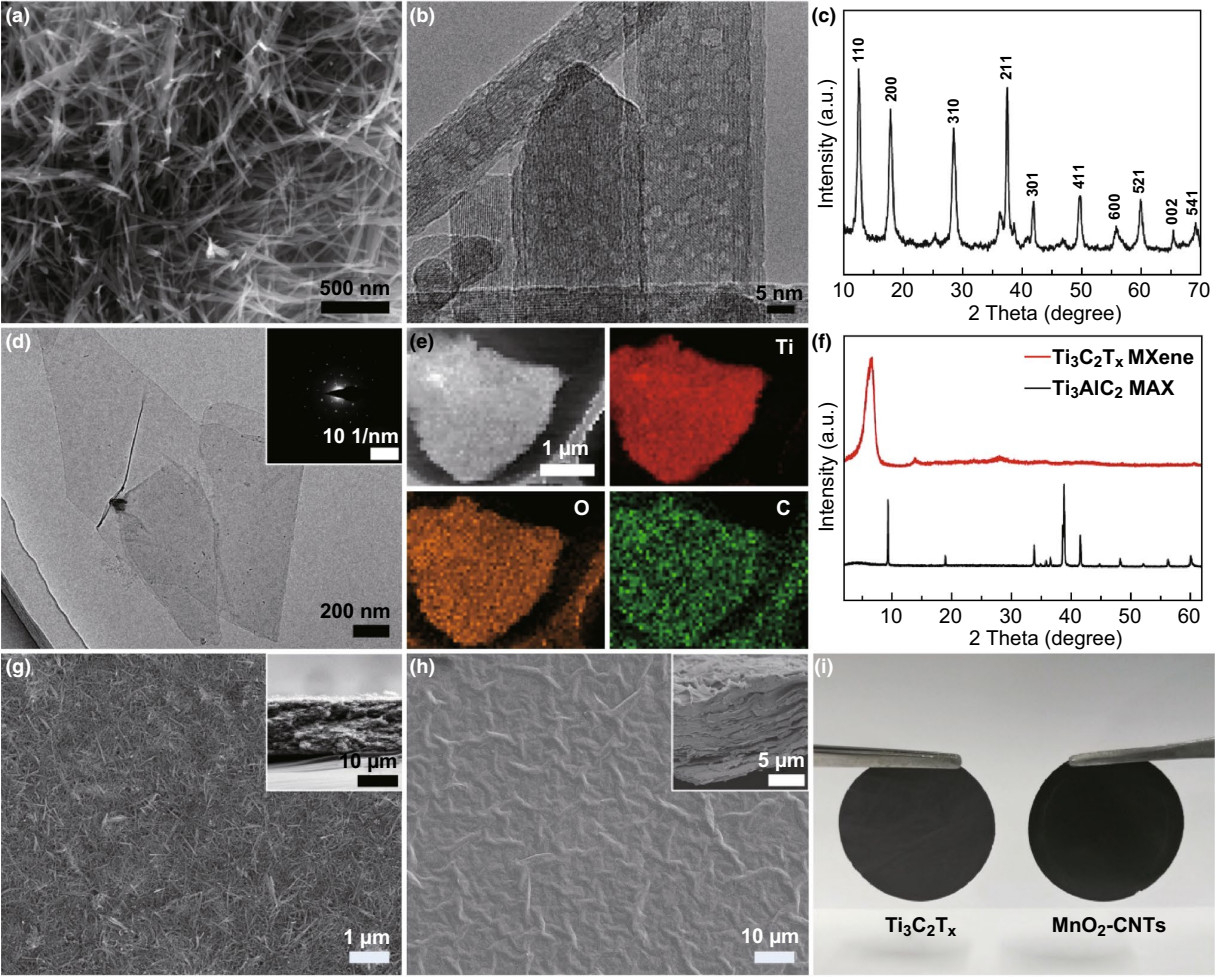
Characterization of the MnO_2_–CNTs battery-type cathode and MXene capacitor-type anode. **a** SEM image, **b** HRTEM image, and **c** XRD pattern of the MnO_2_ NWs. **d** TEM image of the MXene nanosheets, inset is the corresponding SAED, **e** elemental mapping images of the MXene nanosheets, and **f** XRD patterns of MAX and MXene. SEM images of **g** MnO_2_–CNTs and **h** MXene electrodes, insets are the corresponding cross-sectional SEM images, respectively. **i** Photographs of the free-standing MnO_2_–CNTs and MXene electrodes

To prepare the free-standing MnO_2_–CNTs electrodes, MnO_2_ NWs need to be synthesized. Figure 2a shows the SEM image of MnO_2_, which shows the entangled MnO_2_ NWs. The TEM image of MnO_2_ NWs is shown in Fig. 2b. In the TEM image, the lattice fringes can be clearly observed, which illustrates the high crystallization of MnO_2_ NWs. The MnO_2_ NWs have a diameter of 5–25 nm and length of 0.2–2 μm (Fig. S1a). The elemental mapping images of the MnO_2_ NWs (Fig. S1b) clearly show Mn and O. The XRD pattern of the MnO_2_ NWs (Fig. 2c) further confirms that MnO_2_ belongs to the tetragonal α-MnO_2_ phase (JCPDS No. 44-0141) [30, 33]. Before preparing the free-standing MXene electrodes, the highly dispersed MXene solution needs to be obtained. Figure S2 shows the Tyndall scattering effect of the MXene colloid, which illustrates the excellent dispersion of the MXene nanosheets. The two-dimensional lamellar structure of MXene is shown in the TEM image in Fig. 2d. Figure 2d inset shows the selected area electron diffraction (SAED) of the MXene nanosheets, exhibiting a typical hexagonal symmetry diffraction pattern, which demonstrates the single-crystal structure of the MXene nanosheets. Ti, C, and O in Ti_3_C_2_T_*x*_ MXene are clearly observed in the elemental mapping images in Fig. 2e. XRD was conducted to further illustrate the successful preparation of MXene. As shown in Fig. 2f, after etching Al from Ti_3_AlC_2_ MAX, the typical peaks of Ti_3_AlC_2_ MAX disappear, and a new peak at approximately 7°, which belongs to Ti_3_C_2_T_*x*_ MXene, appears [34]. Figure 2g and the inset show the surface and cross-sectional SEM images of the MnO_2_–CNTs cathode, respectively. The thickness of the MnO_2_–CNTs cathode is approximately 12.5 μm. The MnO_2_ NWs are dispersed uniformly in CNTs, which is beneficial for charge transport. The wrinkled structure of the MXene anode with a thickness of approximately 11.5 μm (Fig. 2h inset) is shown in the surface SEM images (Fig. 2h). Figure 2i presents the photographs of the free-standing MnO_2_–CNTs cathode and MXene anode.

**Fig. 3:**
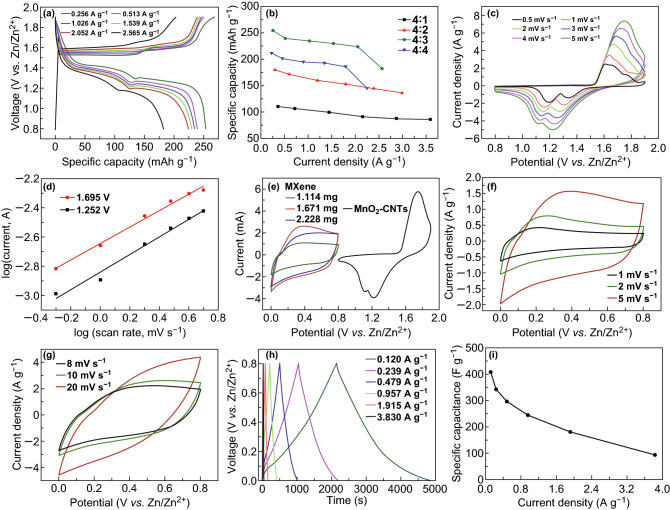
Electrochemical performance of the MnO_2_–CNTs and MXene electrodes. **a** GCD curves with various current densities of the optimum MnO_2_–CNTs electrode. **b** Specific capacitance versus current density of the MnO_2_–CNTs electrodes with various CNTs to MnO_2_ weight ratios. **c** CV curves with various scan rates of the optimum MnO_2_–CNTs electrode. **d** log (*i*) versus log (*v*) plots of the current response at the voltages of 1.695 V and 1.252 V. **e** CV curves of the MXene electrodes (various mass loadings) and MnO_2_–CNTs cathode at a scan rate of 5 mV s^−1^. **f**, **g** CV curves with various scan rates, **h** GCD curves with various current densities, and **i** specific capacitance versus current density of the MXene anode

The typical two-electrode system was used in electrochemical measurements to evaluate the electrochemical performance of the MnO_2_–CNTs electrodes. Figures 3a and S3a–c show the GCD curves of the MnO_2_–CNTs electrodes with different weight ratios. All GCD curves present the clear charge–discharge voltage plateaus. The corresponding relation between the specific capacity and current density of the MnO_2_–CNTs electrodes can be seen in Fig. 3b. When the current density is 0.256 A g^−1^, the specific capacity of the MnO_2_–CNTs electrode with the CNTs to MnO_2_ weight ratio of 4/3 is as high as 254.1 mAh g^−1^, which is much larger than those in previous reports [35–37]. Compared with the other weight ratios, the MnO_2_–CNTs electrode with the CNTs to MnO_2_ weight ratio of 4/3 exhibits the largest specific capacity at the same current density (Fig. S4). This observation can be explained as follows. Because the MnO_2_ has higher theoretical capacity than CNTs, when the content of MnO_2_ in the MnO_2_–CNTs electrode is increased, the corresponding specific capacity is improved. However, the excessive content of MnO_2_ leads to the reduced specific capacity for the intrinsically poor electrical conductivity of MnO_2_. Therefore, there is an optimum CNT to MnO_2_ weight ratio. This phenomenon has been also observed in other studies [38, 39]. Based on the above results and analyses, the MnO_2_–CNTs electrode with a weight ratio of 4/3 was chosen for the cathode of ZIC. To better understand the excellent performance of the MnO_2_–CNTs cathode, electrochemical kinetics was studied in detail based on the CV curves at various scan rates (Fig. 3c). Based on the power law, the relation between the measured current (*i*) and scan rates (*v*) obeys an empirical relation: *i *= *av*^*b*^, where *a* and *b* are the adjustable parameters. From the slope of the log (*i*) versus log (*v*) plot, the value of *b* providing insight into the energy storage mechanism can be obtained. The value of *b* close to 0.5 means a diffusion-controlled process, while the value of *b* near 1 indicates that the electrochemical reaction is dominated by the surface capacitive-controlled process [40]. Figure 3d shows that the *b* values at 1.252 and 1.695 V are 0.60 and 0.56, respectively. These values imply that the corresponding reaction in the MnO_2_–CNTs cathode is mainly controlled by ion diffusion.

It is known that the capacitance balance between the cathode and anode is extremely important to maximize the energy density of the ion capacitors [41, 42]. Thus, the MXene free-standing electrodes with various mass loadings were prepared. Figure 3e shows the CV curves of the MXene electrodes and MnO_2_–CNTs cathode. The capacitance of the MXene electrode with a mass loading of 1.671 mg (0.7 cm^2^) is closest to that of the MnO_2_–CNTs cathode at a scan rate of 5 mV s^−1^. Thus, MXene electrode with this mass loading was used as the anode of ZIC. Next, CV and GCD were investigated to characterize the electrochemical performance of the MXene anode. Figure 3f, g presents the CV curves of the MXene anode. As expected, the CV plots show the rectangular curves, which indicate the ideal capacitive behavior of the anode. The GCD curves in Fig. 3h exhibit good linear potential–time profiles, which further demonstrate the excellent electrochemical performance of the anode. The specific capacitance of the MXene anode evaluated by the above GCD results can be seen in Fig. 3i. The specific capacitance decreases with the increase in current density. When the current density is 0.12 A g^−1^, the specific capacitance of the anode is as high as 408 F g^−1^. The large specific capacitance of the anode avoids the mismatch in capacitance between the cathode and anode of the ZIC.

**Fig. 4:**
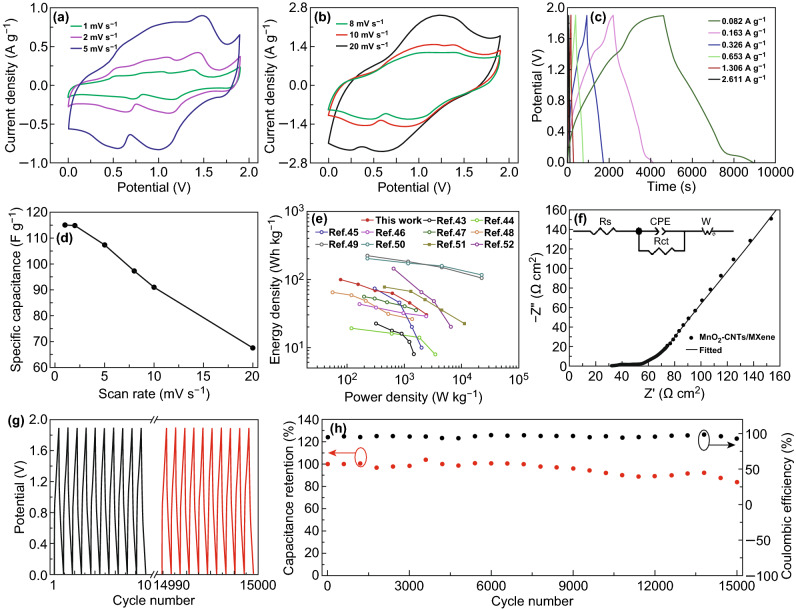
Electrochemical performance of the ZIC in the aqueous liquid electrolyte. **a**, **b** CV curves with various scan rates, **c** GCD curves with various current densities, **d** specific capacitance versus scan rate, **e** energy and power density plot, **f** Nyquist plot (inset is a fitted circuit diagram), **g** GCD curves of the first and last ten cycles, and **h** cycle life and Coulombic efficiency of the ZIC

As a proof of concept, the electrochemical performance of the ZIC in an aqueous liquid electrolyte based on the free-standing MnO_2_–CNTs cathode and MXene anode was investigated. Figure 4a, b shows the CV curves of the ZIC with scan rates from 1 to 20 mV s^−1^. The CV curves exhibit typical redox peaks and large current densities. In Fig. 4c, the well-symmetric GCD curves (current densities from 0.082 to 2.611 A g^−1^) are observed, which further demonstrate the good electrochemical performance of the ZIC. Based on the above CV results, the relation between specific capacitance of the ZIC and scan rate is shown in Fig. 4d. With the scan rate increases from 1 to 20 mV s^−1^, the specific capacitance of the ZIC decreases from 115.1 to 67.5 F g^−1^. Because the GCD curves are approximately linear (Fig. 4c), the power density (*P*) and energy density (*E*) of the ZIC are calculated using the following relation: $P = (V_{\rm{max} } + V_{\rm{min} } )i/2m$, *E *= *Pt*, where *V*_min_ and *V*_max_ represent the lower and upper limits of the voltage window at different current densities, *i* is the discharge current, *m* is the total weight of the cathode and anode, and *t* is the discharge time. The energy density of the ZIC can reach to 98.6 Wh kg^−1^ at the power density of 77.5 W kg^−1^, even the power density of the ZIC is as high as 2480.6 W kg^−1^, and the energy density still retains a high value of 29.7 Wh kg^−1^ (Fig. 4e). The energy and power densities of the ZIC are comparable to those of lithium-ion and sodium-ion capacitors [43–52]. Figure 4f shows the Nyquist plot of the ZIC, and the inset shows the fitted circuit diagram. The equivalent series resistance (ESR) represented by the intercept of semicircle on the real axis (in high-frequency regions) contains the contact resistance between the electrode material and electrolyte, and the internal resistance of the electrode material and electrolyte. The charge transfer resistance (*R*_ct_) affected by the reaction kinetics is reflected by the radius of the semicircle. In low-frequency regions, the slope of the line represents the diffusion resistance of Zn ions in the electrolyte [53]. The ESR and *R*_ct_ of the ZIC are as low as 32.5 and 19.6 Ω, respectively, which implies the fast charge–discharge rate of the ZIC. The cycle life of the ZIC was investigated by GCD. Figure 4g shows the GCD curves of the initial and last ten cycles. As seen in Fig. 4h, after 15,000 GCD cycles at a current density of 5.224 A g^−1^, the capacitance retention of the ZIC is as high as 83.6%, and the Coulombic efficiency is still above 93.3% during cycling. These above results exhaustively demonstrate the excellent performance of the ZIC. To demonstrate the excellent cycle stability of the ZIC, the posttest characterization of the MnO_2_–CNTs cathode and MXene anode was conducted. As shown in Fig. S5a–d, the morphologies and structures after 15,000 GCD cycles have no obvious change, which indicates the excellent stability of the cathode and anode during cycling.

**Fig. 5:**
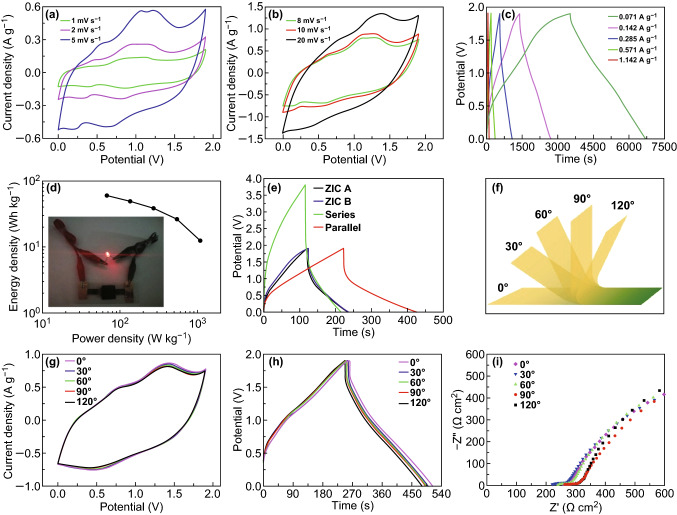
Electrochemical performance and flexibility of the ZIC in the aqueous gel electrolyte. **a**, **b** CV curves, **c** GCD curves, and **d** energy and power density plot (inset shows a LED drove brightly by a single ZIC) of the ZIC. **e** GCD curves of a single ZIC (A, B) and ZICs connected in series and in parallel. **f** Schematic diagram of the flexible display of the ZIC. **g** CV curves at a scan rate of 10 mV s^−1^, **h** GCD curves at a current density of 0.415 A g^−1^, and **i** Nyquist plot of the ZIC bent from 0° to 150°

To illustrate the practical application of the ZIC, the aqueous gel electrolyte was used, and the corresponding electrochemical performance and flexibility were investigated. As shown in Fig. 5a, b, the CV curves of the ZIC also show the high current density and large enclosed area. In Fig. 5c, from the GCD curves of the ZIC, good linear potential–time profiles and long discharge times under certain current densities can be observed. The specific capacitance of the ZIC in an aqueous gel electrolyte is as high as 97.3 F g^−1^ at the scan rate of 1 mV s^−1^ (Fig. S6). Compared with the aqueous liquid electrolyte, owing to the lower conductivity of the aqueous gel electrolyte, the rate capability of the ZIC in the gel electrolyte is slightly worse than that in the liquid electrolyte. The energy and power densities of the ZIC in the aqueous gel electrolyte (Fig. 5d) also exhibit the high values of 67.8 Wh kg^−1^ (59.9 W kg^−1^) and 1085.3 W kg^−1^ (12.4 Wh kg^−1^), respectively. As shown in Fig. 5d inset, a single ZIC can light up a LED brightly. To meet the energy and power needs, two ZICs were assembled in series and in parallel. Figure 5e presents the GCD curves of a single ZIC and ZICs connected in series and in parallel. Two ZICs connected in series exhibit a 3.8 V voltage window with similar discharge times. When the two ZICs are connected in parallel, their discharge time doubles compared to that of a single ZIC. These results obey the basic rule of series and parallel connections of capacitors. Figure 5f illustrates the schematic diagram of the flexible display of the ZIC in the aqueous gel electrolyte. The CV and GCD curves of the ZIC with bending angle from 0° to 120° are shown in Fig. 5g, h. It can be concluded that the specific capacitance of ZIC changes little during bending. (The capacitance retention calculated by CV results remains almost 94.6% when bends up to 120° (Fig. S7).) The Nyquist plots of the flexible ZIC keep almost identical during bending except for a slight increase in ESR (increased from 218.7 Ω (0°) to 269.9 Ω (120°)), as shown in Fig. 5i. The above results imply that the designed ZIC in the aqueous gel electrolyte possesses excellent electrochemical performance and flexibility.

## Conclusions

A new ZIC was successfully prepared by assembling free-standing MnO_2_–CNTs battery-type cathode and MXene capacitor-type anode in aqueous liquid or aqueous gel electrolytes. The ZIC assembled in the aqueous electrolyte avoids the inflammable, volatile, and toxic safety issues. Similar to the lithium-ion and sodium-ion capacitors, the ZIC stores energy using the battery-type and capacitor-type energy storage mechanisms. As free-standing electrodes without the binder are adopted, the mass and volume of the ZIC are considerably lower. These above-mentioned reasons indicate the excellent energy storage performance of the ZIC. As expected, the ZIC exhibits a high specific capacitance of 115.1 F g^−1^ (scan rate of 1 mV s^−1^), a high energy density of 98.6 Wh kg^−1^ (power density of 77.5 W kg^−1^), and a high power density of 2480.6 W kg^−1^ (energy density of 29.7 Wh kg^−1^). Even after 15,000 GCD cycles, the ZIC exhibits a high capacitance retention of ~ 83.6% of its initial capacitance, and during the cycles, it has a high Coulombic efficiency of above 93.3%. In aqueous gel electrolyte, the ZIC also exhibits excellent performance. This study provides an effective way to design an ion capacitor with high energy and power densities, excellent cycle stability, long life, and high safety for the next-generation energy storage devices.

## Electronic supplementary material

Below is the link to the electronic supplementary material.

Supplementary material 1 (PDF 708 kb)
