# Supplementary material for: A New Free-Standing Aqueous Zinc-Ion Capacitor Based on MnO2–CNTs Cathode and MXene Anode
Source: Nanomicro Lett. 2019 Aug 26;11:70. doi: 10.1007/s40820-019-0301-1 (PMC7770692; doi:10.1007/s40820-019-0301-1)
Supplement: Supplementary file 1 — Supplementary material 1 (PDF 708 kb) [file 40820_2019_301_MOESM1_ESM.pdf]

Supporting Information for

A New Free-standing Aqueous Zinc-ion Capacitor Based on MnO_2-CNTs Cathode and MXene Anode

Siliang Wang[1, 2], Qiang Wang[1, 2], Wei Zeng[1, 2, *], Min Wang[1, 2], Limin Ruan[1, 2], Yanan Ma[3, *]

[1]Key Laboratory of Intelligent Computing & Signal Processing, Ministry of Education, Anhui University, No. 3 Feixi Road, Hefei 230039, Anhui Province, People's Republic of China

[2]National Engineering Research Center for Agro-Ecological Big Data Analysis & Application, School of Electronics and Information Engineering, Anhui University, No. 111 Jiulong Road, Hefei 230601, Anhui Province, People's Republic of China

[3]School of Sciences, Hubei University of Automotive Technology, No. 167 Checheng West Road, Shiyan 442002, Hubei Province, People's Republic of China

*Corresponding authors. E-mail: youfmail@163.com (Wei Zeng); mayn@huat.edu.cn (Yanan Ma)

Supplementary Figures

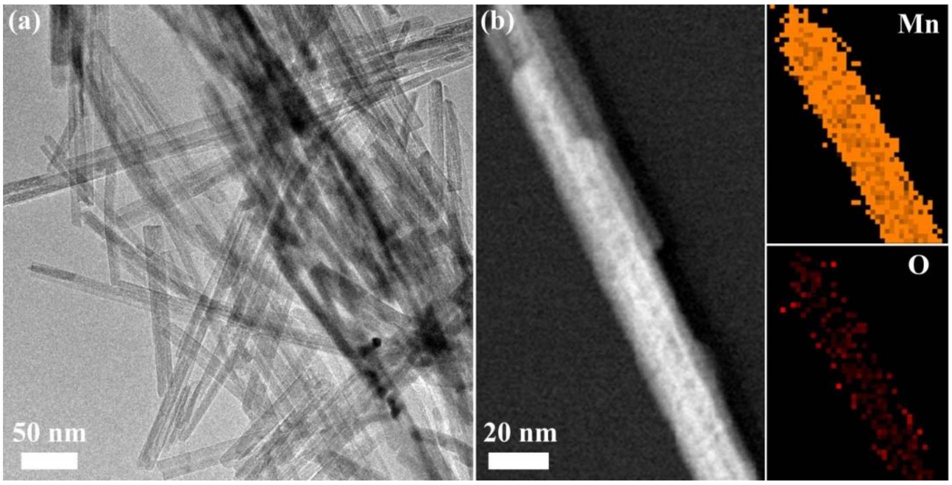

Fig. S1 Characterization of the MnO_2 NWs. **a** TEM image and **b** elemental mapping images of the MnO_2 NWs

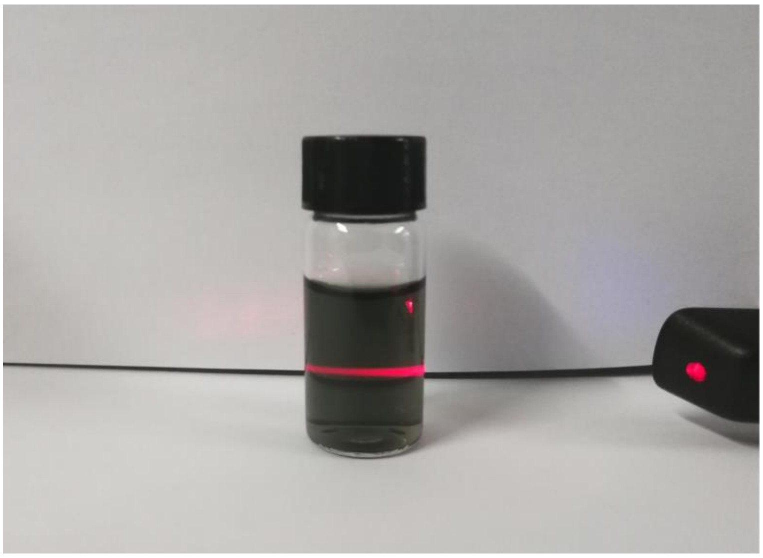

Fig. S2 Tyndall effect of the MXene colloid

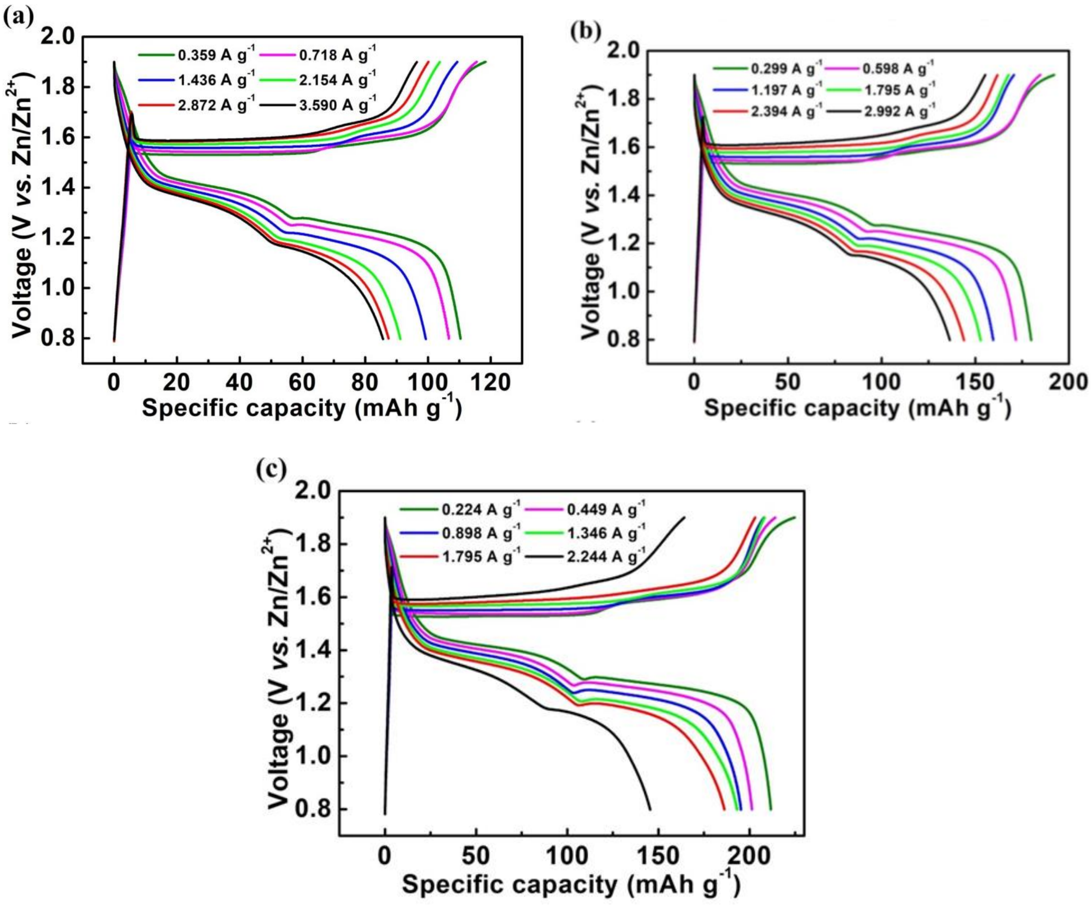

Fig. S3 GCD curves of the MnO_2-CNTs electrodes with various CNTs to MnO_2 weight ratios. CNTs to MnO_2 weight ratios of **a** 4/1, **b** 4/2, and **c** 4/4

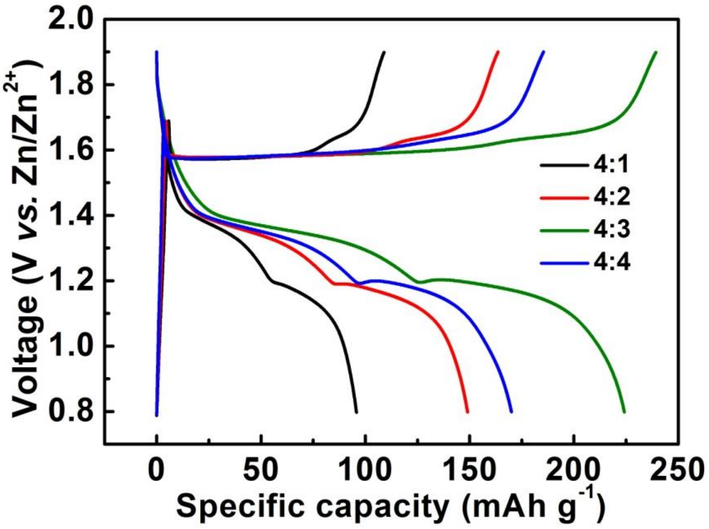

Fig. S4 GCD curves of the MnO_2-CNTs electrodes with various CNTs to MnO_2 weight ratios at a current density of 2.052 A g^{-1}

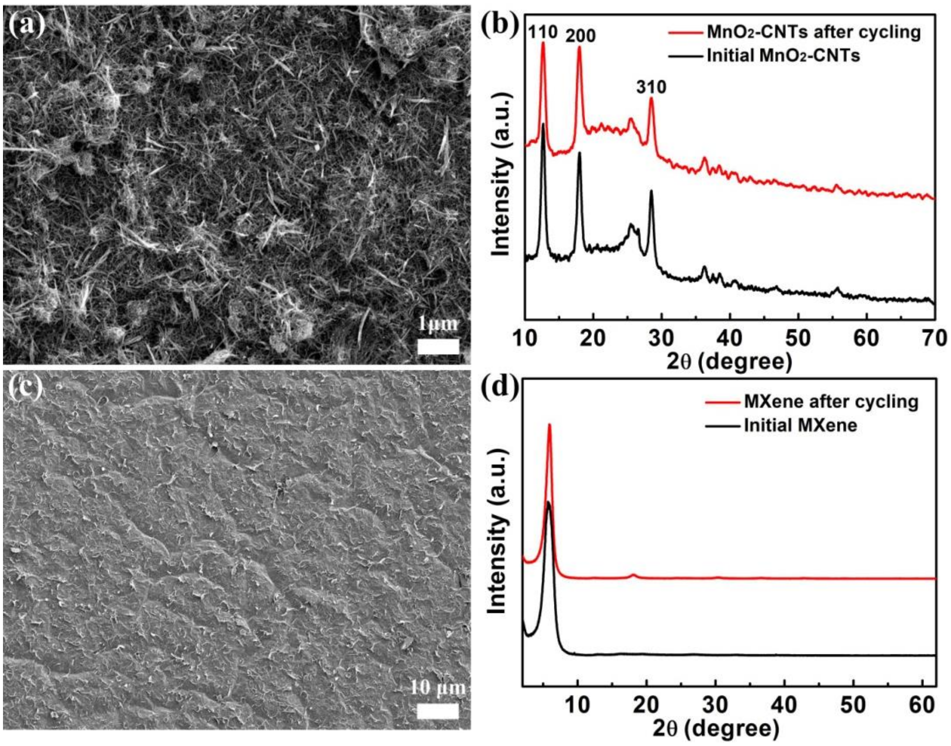

Fig. S5 Characterization of the MnO_2-CNTs cathode (charged to 1.9 V) and MXene anode after 15,000 GCD cycles. **a** SEM image and **b** XRD pattern of the MnO_2-CNTs cathode after cycling. **c** SEM image and **d** XRD pattern of the MXene anode after cycling

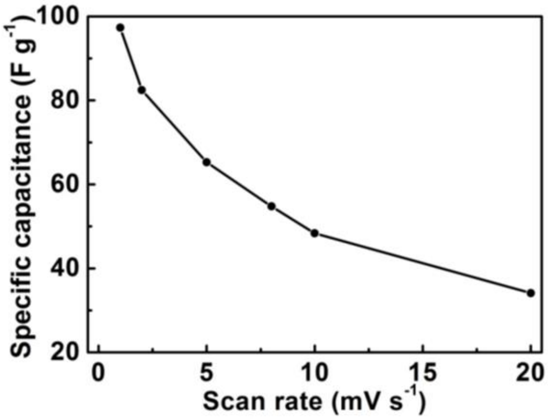

Fig. S6 Specific capacitance *vs.* scan rate of the ZIC in the aqueous gel electrolyte

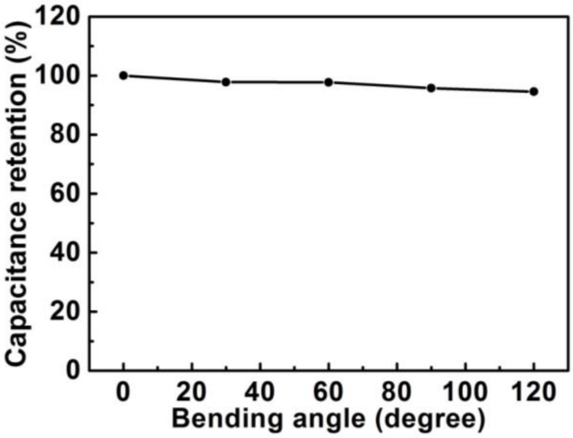

Fig. S7 Capacitance retention *vs.* bending angle of the ZIC in the aqueous gel electrolyte
